# Developing Leadership: The Integrative Approach of Pro●Leader Intervention Program

**DOI:** 10.3390/bs15050601

**Published:** 2025-04-30

**Authors:** A. Rui Gomes, Catarina Morais

**Affiliations:** 1 Psychology Research Centre, School of Psychology, University of Minho, 4710-057 Braga, Portugal; rgomes@psi.uminho.pt; 2 Research Centre for Human Development, Faculty of Education and Psychology, Universidade Católica Portuguesa, 4169-005 Porto, Portugal

**Keywords:** leadership training, Leadership Efficacy Model, leadership styles, leadership plan, leadership cycles

## Abstract

The impact of leaders on organizational success has been widely documented, and consequently, the leadership training industry has expanded over the past decades. However, this investment has not consistently translated into better leaders. Leadership intervention programs have received notable criticism, including (a) lacking individualization (‘one size fits all’ approach), (b) an emphasis on pre-defined skills, and (c) insufficient consideration of context, disregarding other members’ and organizations’ characteristics. This paper introduces and discusses Pro●Leader, an intervention program based on the Leadership Efficacy Model (LEM), which aims to promote leadership through a comprehensive approach to training. In essence, LEM proposes that leadership efficacy increases if leaders are congruent in how they intend to exert leadership (conceptual cycle of leadership) and how they indeed implement the leadership (practical cycle of leadership), considering also the leadership behaviors they display when exerting influence (leadership styles), as well as their own, team members’, and contextual characteristics (antecedent factors). The Pro●Leader intervention program is structured according to the LEM and trains participants across these three components: leadership cycles, styles, and antecedent factors.

## 1. Introduction

The role of leaders in organizational success is widely recognized. Leadership is a process of influencing others towards common goals, promoting organizational productivity and profit and, consequently, competitive advantages (Hogan & Kaiser, 2005; Kaiser et al., 2008; Voon et al., 2010; Yahaya & Ebrahim, 2016). By the end of the 2000s, in the United States alone, leadership training was a $14 billion industry (O’Leonard & Loew, 2012) and in 2018, Kellerman (2018) argued for an estimation of around $50 billion being spent on leadership training. However, the investment in leadership training and development has not consistently produced better leaders (Kaiser & Curphy, 2013). A report released by McKinsey & Company showed that two-thirds of the 500 senior executives interviewed considered leadership development as the most urgent concern of human capital (Gurdjian et al., 2014). If companies are investing so heavily in leadership development, why does it remain such a pressing issue? Does this suggest that leadership development programs are failing to enhance leadership efficacy?

### Do Organizations Need Leadership Development Programs?

Previous research has shown that leadership interventions are effective in a wide range of outcomes, such as promotions and assuming leadership roles, increasing leadership knowledge and technical performance, and reducing organizational absenteeism (Dannels et al., 2008; Day et al., 2010; Geerts et al., 2019; Straus et al., 2013; ten Have et al., 2013). A few meta-analyses of such research summarize the results. For example, Cummings et al. (2021) conducted a meta-analysis examining 93 studies with nurses (44 correlational studies and the remaining intervention studies) and concluded that participating in leadership interventions had a positive effect on the development of relational leadership styles. Moreover, the authors highlighted five categories of factors contributing to nursing leadership across studies: experience and education (e.g., professional development opportunities and performance feedback), leaders’ traits and personal characteristics (e.g., age, emotional intelligence, adjustment), relationship with work, role in the organizational setting (lower seniority), and organizational context. When examining only the studies where an intervention occurred (*n* = 49), in 82% of them, self-reported leadership increased post-intervention, and in 20% of them, it was reported that there was an increase in observed leadership practices. However, it is important to note that few experimental intervention studies (i.e., those including a control group) were included in this meta-analysis.

Avolio et. al. (2009) took a step further and conducted two meta-analytic reviews on leadership interventions and their impact. The study included different theoretical frameworks for leadership development (e.g., trait theories, transformational leadership), different interventions (training, assignment, scenario, or others), methodologies (experimental and quasi-experimental, lab and field studies), and organizations (public and private). The overall results support the idea that leadership interventions are effective and contribute to positive outcomes. However, the studies included in this review were notably heterogeneous, as the authors noted, and therefore, their effects need to be carefully considered. More specifically, two major concerns were highlighted.

First, authors compared effect sizes produced by studies that tested leadership interventions vs. other experimental methodologies (e.g., using scenarios or vignettes). While effects were generally similar, they were slightly weaker for leadership interventions compared to other experimental studies (Avolio et al., 2009). One possible explanation proposed by the authors is that other laboratory studies did not occur in the real organizational setting, and, therefore, other factors impacting leadership effectiveness were controlled for, which may have increased effect sizes. The results may also have depended on the outcome measure chosen to evaluate its efficacy, as developmental changes may be more difficult to impact (Avolio et al., 2009).

A second important moderating effect refers to the theory behind the different studies included. More specifically, Avolio et al. (2009) compared three groups of leadership theories: “traditional theories” (e.g., Trait and Contingency Theories), which dominated leadership research until the late 1970s; “newer theories” (e.g., charismatic, inspirational, transformational, and visionary leadership theories); and “Pygmalion based leadership” (e.g., self-fulfilling prophecy effects). These were compared across different types of outcomes: affective (e.g., satisfaction/enjoyment), cognitive (e.g., level of idea generation or confidence), and behavioral (e.g., performance measures of behaviors). The Pygmalion-based studies showed an overall larger effect than the other two theory-based studies, and studies that designed interventions based on “newer” theories showed larger effects on affective and cognitive outcomes when compared to “traditional” theories, and the opposite pattern was found for behavioral measures.

So, if research appears to show that leadership interventions enhance positive results, and organizations continue to invest in leadership training, why is there still a concern about the state of leadership development today? Avolio et al. (2009) meta-analysis included 200 studies over a century of research, but this is not a representative sample of the research conducted on leadership (Avolio et al., 2009; Kaiser & Curphy, 2013), suggesting that an important proportion of studies, which may have yielded no effects or negative effects of leadership development, were not considered because they were not published (cf. Siegel et al., 2022 for a review on publication bias in organizational psychology). Moreover, of the studies considered in the meta-analysis, around two-thirds showed a positive outcome. However, these were largely affective and cognitive outcomes, with relatively few focusing on behavior or actual performance results (Kaiser & Curphy, 2013). The remaining one-third of the interventions reported no positive outcomes. Given that published studies are often more rigorously designed than the remaining interventions, the actual failure rate may be higher than 1:3 (Kaiser & Curphy, 2013).

Some criticism has been made of leadership intervention programs, which may help explain the failure rate and, consequently, the leadership efficacy. The first concerns the lack of rigorous impact evaluation. Leadership interventions offered in the industry are rarely evaluated, or, when evaluated, they often rely only on participants’ feedback (Alimo-Metcalfe & Lawlor, 2001; Gurdjian et al., 2014; Kaiser & Curphy, 2013; Kellerman, 2012). The questions raised regarding leadership interventions’ effectiveness have increased pressure for impact demonstration so a return on investment can be established (Geerts et al., 2019). More research on the impact of leadership interventions on behavioral change is needed, as well as an assessment of its long-term effects. Second, most well-known leadership interventions are pre-defined and skill-focused (Lega et al., 2017), and it is very difficult, due to the complexity of leadership processes, to argue that “one size fits all” interventions would be adequate to promote behavioral change in different organizational settings. Tailoring interventions to meet individuals’ needs increases the likelihood of achieving better results (Melton & Brooks, 2022; Richter et al., 2015). Moreover, it is key to identify one’s deep thoughts, feelings, beliefs, and assumptions about behavior to change it (Gurdjian et al., 2014), which will be difficult to achieve without an individualized plan of intervention. A third, and arguably the most significant, criticism is that many interventions fail to adequately consider contextual factors. This ‘one size fits all’ idea is not only related to the content of interventions themselves, but also to the assumption that by developing a certain set of skills, leaders will be more effective, without considering the organizational setting, its culture, values, ethical climate, working conditions, or strategy (Bricon-Souf & Newman, 2007; Cummings et al., 2021; Gurdjian et al., 2014; Lord et al., 2013). Overlooking contextual factors also excludes the involvement of followers in leadership activities, neglecting teams’ needs and characteristics (Kaiser & Curphy, 2013). When leadership development is disconnected from the real-world context, it becomes harder for participants to apply new knowledge and skills in their real work environment. This detachment from the real work setting is also an important criticism of a significant amount of leadership programs, which operate as a “closed system” and disregard how feedback loops within an organization can help leaders better manage the consequences of their actions (cf. Day & Dannhäuser, 2024 for a review).

In sum, while leadership interventions can produce positive outcomes, their design and implementation generally fail to individualize leaders’ needs and the context where leadership occurs, namely followers’ and organization characteristics, which may be the main factors contributing to leadership development interventions not reaching their full potential. In other words, leadership programs need to be tailored and theoretically and evidence-based, adapted to the specific needs of their participants and organizations where leadership occurs (Day et al., 2014; Galuska, 2014). Thus, this paper presents the Pro●Leader intervention program, which is an intervention based on the Leadership Efficacy Model (LEM) that aims to promote leadership efficacy, considering not only leaders’ skills but their relationship with followers’ and organizations’ specific needs, focusing not only on the leader but considering also their surroundings, therefore taking a more comprehensive approach. The designation “Pro●Leader” tries to capture the idea of “Professional Leaders”, providing training to augment the leadership expertise of participants.

## 2. The Leadership Efficacy Model: A Framework for Leadership Development

To fully understand the Leadership Efficacy Model (Gomes, 2014, 2020), it is first important to clarify the concept of leadership itself. A general consensual definition of leadership refers to a process through which leaders influence others with the aim of guiding or facilitating relationships within groups and organizations (Yukl, 2013). What this definition does not encompass is the consequences of leadership. Thus, Gomes (2023, 2024) argued that it is important to consider the outcomes in the definition itself and, therefore, leadership development should focus on enhancing the benefits for those involved in the process (both leaders and followers). Therefore, the author suggested a definition of leadership that refers to it as a process of influence exerted on others that is sustained on common and positive ideals that lead all involved to a better situation than the one that preceded the leader’s influence. This is intrinsically linked to the concept of leadership efficacy, which has been broadly defined as the impact and consequences of leadership, i.e., the outcomes of the leadership process that can be measured at the individual, group, or organizational level (Kaiser et al., 2008; cf. Hannah et al., 2008 for a review). Following this definition, leadership cannot be separated from its impacts. The “status” of leadership, in fact, requires the simultaneous consideration of three factors: (a) the most known dimension of the influence exerted by the leader onto others (individuals or members of groups/teams, organizations, communities, or even societies), (b) the need to formulate common and positive ideals for all those involved in the leadership phenomenon, and (c) the need to achieve a better scenario than the one predating the leader’s influence.

**Figure 1:**
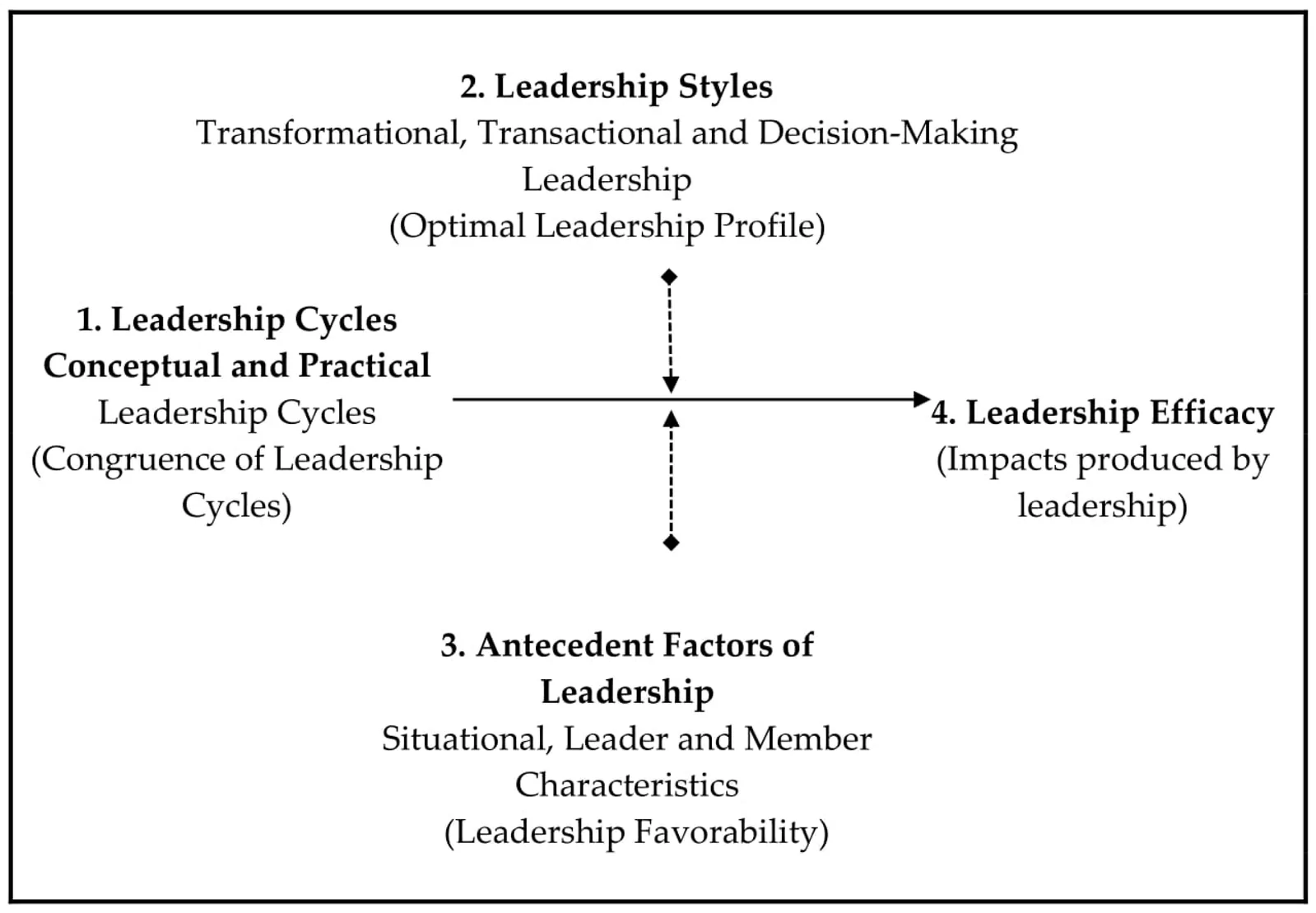
Leadership efficacy model (retrieved from Gomes, 2020).

The Leadership Efficacy Model (LEM; Gomes, 2014, 2020) integrates different approaches to leadership (trait, behavioral, and contingency leadership theories) in a comprehensive framework that establishes and combines distinct factors that influence leadership efficacy. In a nutshell, this theoretical approach is based on the assumption that leadership efficacy increases if leaders are congruent in how they intend to exert leadership (conceptual cycle of leadership) and how they actually implement the leadership (practical cycle of leadership), considering also the behaviors displayed by leaders when exerting influence (leadership styles), as well as their own, team members’, and contextual characteristics (antecedent factors). Specifically, this framework proposes that three different sets of factors influence leadership efficacy: (1) leadership cycles, (2) leadership styles, and (3) antecedent factors of leadership (cf. Figure 1).

The leadership cycles component refers to the congruency between leaders’ ideas and actions. More specifically, it is proposed that a leadership cycle is composed of (a) leadership philosophy, i.e., leaders’ concept of how leadership should be (one’s values, assumptions, and beliefs about leadership); (b) leadership practice, i.e., the actions that reflect leadership philosophy (how leadership should be implemented); and (c) leadership criteria, i.e., indicators used to evaluate the implementation of philosophy and practice of leadership. This leadership cycle occurs at two different levels: a conceptual level (i.e., how the leader thinks their leadership should be in terms of philosophy, practice, and criteria) and a practical cycle (i.e., how the leaders implement the conceptual cycle in a certain real scenario) (Gomes, 2020). According to the model, the greater the degree to which leaders’ philosophies and ideas (conceptual cycle) are in line with how they actually implement their leadership (practical cycle), the better their leadership efficacy (Gomes, 2020, 2023). Thus, leaders should be aware of the need to establish a congruence between the conceptual and practical cycles, defining their most important ideas, behaviors, and evaluation indicators.

The second factor refers to the specific behaviors that leaders adopt when exerting their leadership. Based on previous literature that has tested psychological measures of leadership behaviors (Gomes et al., 2021; Gomes & Resende, 2014), the LEM highlights three different styles of leadership (transformational leadership, transactional leadership, and decision-making leadership) and nine leadership behaviors divided into transformational leadership (vision, inspiration, instruction, individualization, and support), transactional leadership (positive and negative feedback), and decision-making leadership (active management and passive management). Transformational leadership relates to the leaders’ ability to promote change in their followers’ attitudes, beliefs, and values towards the goals of the group/organization, so that leaders’ vision becomes internalized (cf. Bass, 1985; Bass & Avolio, 1994). In order to do so, leaders display a positive and enthusiastic vision, inspire followers to make continuous efforts to achieve success, provide instructions to help them develop skills, and attend to the individual needs of each follower by supporting their interests and wellbeing (Gomes, 2020). Transformational leadership has been widely researched (Bass, 1990; Burns, 1978; Judge & Piccolo, 2004; Young et al., 2021), representing a major topic of analysis when it comes to studying the effects produced by leadership (Ng, 2017; Stock et al., 2023). The popularity of this form of leadership remains intact even though new forms of leadership have been developed. However, these new proposals, such as moral and ethical leadership, authentic leadership, and servant leadership, appear to overlap significantly with transformational leadership (Banks et al., 2018; Hoch et al., 2018; Mayer et al., 2012). On the other hand, the transactional style of leadership is based on exchange systems, in a “negotiation” between what leaders want and what team members provide (cf. Avolio & Bass, 2004). More specifically, when using this approach, leaders tend to respond to their followers’ actions and performance, managing them using positive and negative feedback (Gomes, 2020) to establish and promote the desired behaviors. The third and final style is related to how leaders make decisions or, more specifically, how they manage their power (Northouse, 2021). Decision-making leadership behaviors differentiate between active and passive management, referring, respectively, to whether leaders tend to share or not share leadership power and whether they involve followers in decision-making related to the teams’ functioning (Gomes & Resende, 2014).

The novelty behind leadership styles in the Leadership Efficacy Model is the argument that leadership styles should be combined to build an optimal profile of leadership (Gomes, 2020). Specifically, an optimal profile of leadership is achieved when leaders use transformational behaviors (i.e., vision, inspiration, instruction, individualization, and support), positive feedback from transactional leadership, and active management from decision-making leadership (decentralizing decision-making), while reducing the occurrence of negative feedback from transactional leadership and passive management from decision-making leadership. The main assumption is that the positive combination of these behaviors (optimal profile) will augment leadership efficacy by maximizing leadership cycles.

Finally, the third and final dimension of LEM refers to the antecedent factors of leadership. This dimension comprises a set of factors that can impact leadership efficacy, namely leaders’ characteristics, followers’ characteristics, and situational characteristics (cf. Gomes, 2020). Broadly, these three dimensions include personal and mental characteristics of both leaders and members (e.g., age, gender, personality, coping strategies, competence levels, etc.), as well as the characteristics of the context where leadership occurs (e.g., type of organization, working conditions, responsibilities given to the leader, etc.) (cf. Fiedler, 1993; Gomes, 2020). The overall idea is that leadership does not occur in a vacuum but is instead a dynamic process that needs to adapt and be adapted by the circumstances, thereby improving situational favorability. In other words, the better the match between leaders’ and followers’ characteristics, the higher their competencies to perform, and the better the conditions provided by the context, the higher the leadership efficacy.

In conclusion, the Leadership Efficacy Model provides a theoretical framework for leadership development by considering holistically the process of leadership, emphasizing that in order to increase the efficacy of leadership, a multitude of factors need to be considered: the leadership cycles (by achieving a congruence between conceptual and practical cycles), the leadership behaviors (by assuming the optimal profile of leadership), and the antecedent factors of leadership (by considering the characteristics of the leaders, the followers, and the context). Accordingly, the Pro●Leader intervention program is a proposal of leadership development based on the Leadership Efficacy Model.

## 3. Leadership Development: Pro●Leader Intervention Program

The Pro●Leader intervention program is organized according to the LEM proposal, training participants in the three components of the model: leadership cycles, leadership styles, and antecedent factors of leadership.

The program is based on five core ideas that characterize the way intervention is delivered to participants. First, the intervention program is aligned with the notion of life skills training, including, sequentially, activities that are part of the four stages of life skills acquisition (cf. Gomes & Resende, 2020): (a) motivation: activities that aim to guarantee that participants mobilize attention and personal interest for the development of leadership skills; (b) learning: activities that aim to enhance participants’ understanding of how leadership works and how it can be used properly; (c) automatization: activities that aim to provide participants the opportunity to use the life skill in a real-setting situation; and (d) transference: activities that allow participants to understand how to use the life skills in different situations, preferably from distinct life contexts. In practical terms, the program intends to stimulate the interest and knowledge of participants regarding leadership and to develop their leadership skills by providing structured opportunities that facilitate the automatization and generalization of leadership skills to participants’ contexts.

Second, the program is theoretically grounded by bringing together aspects related to the philosophy, practice, and criteria of leaders (e.g., Gould et al., 2017; Lyle & Cushion, 2016), the behaviors of leaders (e.g., Antonakis, 2012; Sosik & Jung, 2018), and the characteristics of leaders, team members, and context where leadership occurs (e.g., Northouse, 2021). These distinct factors allow participants to understand the key points related to the efficacy of leadership, namely the cognitions, beliefs, and principles of leaders (leadership cycles); the actions of leadership (leadership behaviors); and the contingencies of the exercise of leadership (antecedent factors of leadership related to the characteristics of leaders, team members, and context where leadership occurs). Of course, many other factors are involved in the leadership activity, but this set of factors included in the LEM is involved in the impact produced by leadership.

Third, the program should be delivered to small groups (maximum of 15 participants) by two practitioners with professional certification in psychology. This participant/practitioner ratio aims to maximize the likelihood that all participants will have the opportunity to acquire leadership skills in an individualized manner and receive detailed feedback. This is evident, as all participants develop, discuss, and implement a leadership plan during the training, receiving feedback from both practitioners and peers. Additionally, this practitioner-to-participant ratio enhances the potential for participants to progress through the four stages of life skills, as previously described.

Fourth, the program includes participants from different professional contexts, thus reinforcing that the leadership training is applicable to all participants (due to the general theoretical background of the LEM). It also achieves specificity as participants acquire leadership skills relevant to their life contexts. There are no prerequisites to enter the program, so participants may have varying levels of experience in leadership roles, or they may simply be interested in improving their leadership knowledge and skills. The primary goal of the program is to empower participants with the knowledge and skills needed to enhance their leadership capabilities.

Fifth, the program includes opportunities for data collection to evaluate its efficacy. Specifically, data will be collected before the program begins (Phase 1), immediately after it ends (Phase 2), and three to four months after the last session (Phase 3). The third data collection phase allows participants sufficient time to apply their leadership plans developed during the Pro●Leader intervention program to their specific life contexts.

In sum, several factors contribute to leadership efficacy. It is evident that including all of these factors in a single intervention program is challenging, if not impossible. However, the Pro●Leader intervention program incorporates key factors that enhance leadership efficacy and, by extension, participants’ ability to lead.

The Pro●Leader intervention program is organized into seven parts (cf. Table 1): (1) pre-intervention evaluation; (2) leadership behaviors (corresponding to the leadership styles of the LEM): participants are trained in different leadership behaviors; (3) leadership plan (corresponding to the leadership cycles of the LEM): participants are asked to define their own leadership philosophy, practice, and criteria (leadership cycles) and formulate an individual leadership plan; (4) leadership plan adjustment (corresponding to the antecedent factors of leadership of the LEM): participants reflect on how their own characteristics, their team members’ characteristics, and their organizational context can influence their leadership and are encouraged to adjust their leadership plan accordingly; (5) training of the leadership plan: participants assume their own leadership plans by using a roleplay methodology to recreate, as much as possible, their scenario of leadership; (6) post-intervention evaluation; and (7) follow-up intervention evaluation. In the following sections, we describe each part of the Pro●Leader intervention program in more detail.

### 3.1. Intervention Evaluation (Parts 1, 5, and 6)

Testing the efficacy of the intervention is crucial to facilitate progress in leadership knowledge and guide future adjustments to the training program. The Pro●Leader intervention program includes evaluations conducted before and after the intervention (cf. Table 1: Parts 1, 6, and 7). The measures used to evaluate efficacy should be selected according to the interests of each professional or researcher; however, in general, it is important to include two types of measures.

The first relates to leadership and is specifically designed to evaluate the leadership cycles, the leadership styles, and the antecedent factors of leadership (cf. Gomes et al., 2022). For example, for the LEM, there are available measures of the leadership cycles (e.g., Leadership Cycles Questionnaire; Gomes et al., 2022), leadership styles (e.g., Multidimensional Scale of Leadership; Gomes et al., 2021), and antecedent factors of leadership (e.g., Leadership Antecedent Factors Questionnaire; Gomes et al., 2022). The data collected from these instruments can be shared with participants to increase their understanding of their leadership skills and support their leadership plan developed throughout the program, namely when they think about their leadership styles and behaviors (Part 3.3.), their leadership cycles (Part 3.4.), and factors that influence the exercise of leadership (Part 3.5.).

The second type of measure relates to the impacts produced by the participants’ leadership. In this last case, it is important to evaluate the implicit impacts produced by leadership (psychological, relational, and social impacts of leadership) but also the explicit impacts produced by leadership (behavioral impacts of leadership in terms of actions/achievements assumed by team members) (Gomes, 2024). It is also recommended that measures be collected from the perspective of participants and from the individuals who interact with them (e.g., followers, collaborators, team members, etc.), which helps reduce the common method bias (Podsakoff et al., 2012). Nonetheless, it is evident that self-evaluation from participants is easier to collect before and after the Pro●Leader intervention program than external evaluation.

### 3.2. Program Presentation (Part 2)

The program is presented in the first session (included in Part 1 of the program; Table 1) by informing participants about the goals, structure of training, and evaluation system. Participants are divided into three groups, and they must choose a name and motto for their group (to reinforce team identity and facilitate various training activities) (cf. Hoult et al., 2024; Slater et al., 2019).

### 3.3. Leadership Styles and Behaviors (Part 2)

**Figure 2:**
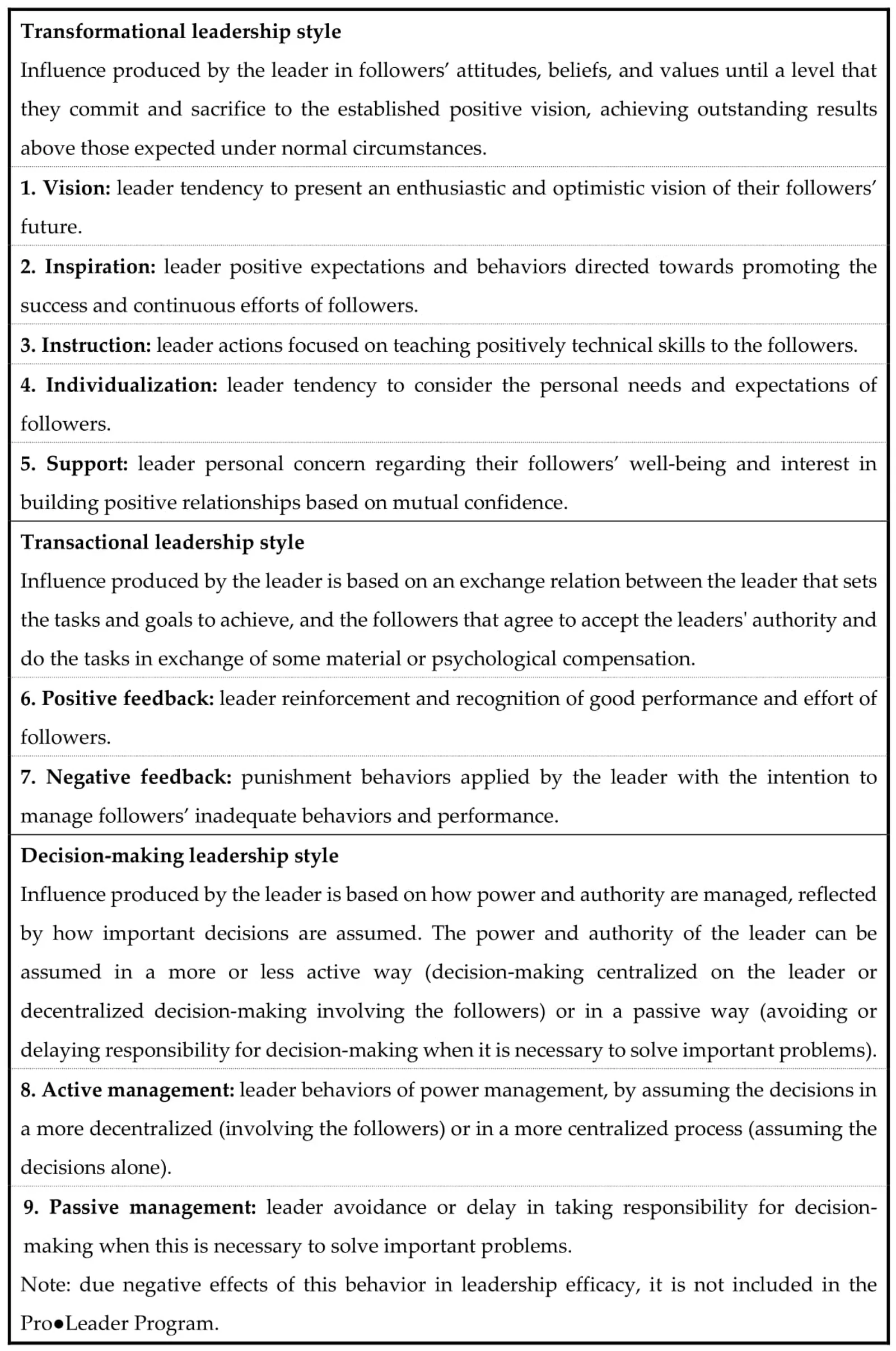
Pro●Leader intervention program: leadership styles and leadership behaviors.

**Figure 3:**
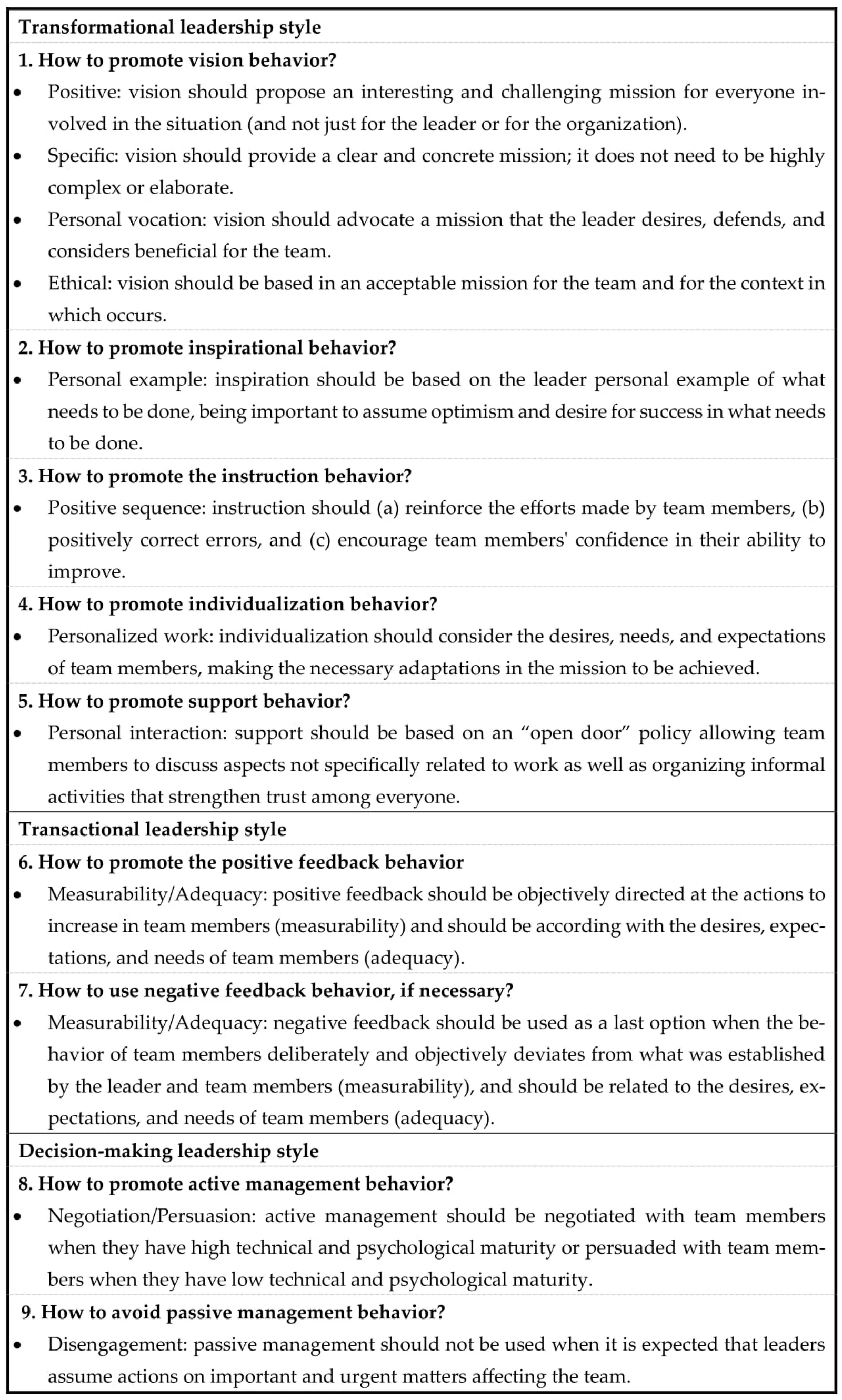
Pro●Leader intervention program: promoting leadership behaviors.

Part 2 of the program is dedicated to educating and training participants in using eight leadership behaviors (cf. Table 1). Starting the program with a focus on leadership styles and behaviors is essential because participants need to master the eight behaviors when they are asked to later formulate the leadership plan. Thus, participants are first introduced to transformational, transactional, and decision-making leadership, along with the respective behaviors of each style (see Figure 2). Then, the concept of an optimal leadership profile is explored. This includes five transformational leadership behaviors (i.e., vision, inspiration, instruction, individualization, and support), positive feedback from transactional leadership behaviors, and active management from decision-making leadership. It is important to clarify that passive management is not trained in the Pro●Leader intervention program and that negative feedback is trained only under highly controlled conditions. Participants are informed that negative feedback should be used only when followers display unacceptable behaviors that violate previously agreed-upon standards. It is also explained that these behaviors will be important to help participants to set their leadership plan, meaning they will use the leadership behaviors to build their leadership cycles (what is their leadership philosophy, practice, and criteria?) and to adjust the cycles to the antecedent factors of leadership. In this way, participants will be aware that later in the program they will select the most useful behaviors to set their leadership plan. Finally, in an open group discussion with all participants, they discuss how to use leadership behaviors in ways that enhance leadership efficacy. At the end of this discussion, practitioners present potential strategies that can maximize the use of each leadership behavior (Figure 3).

**Figure 4:**
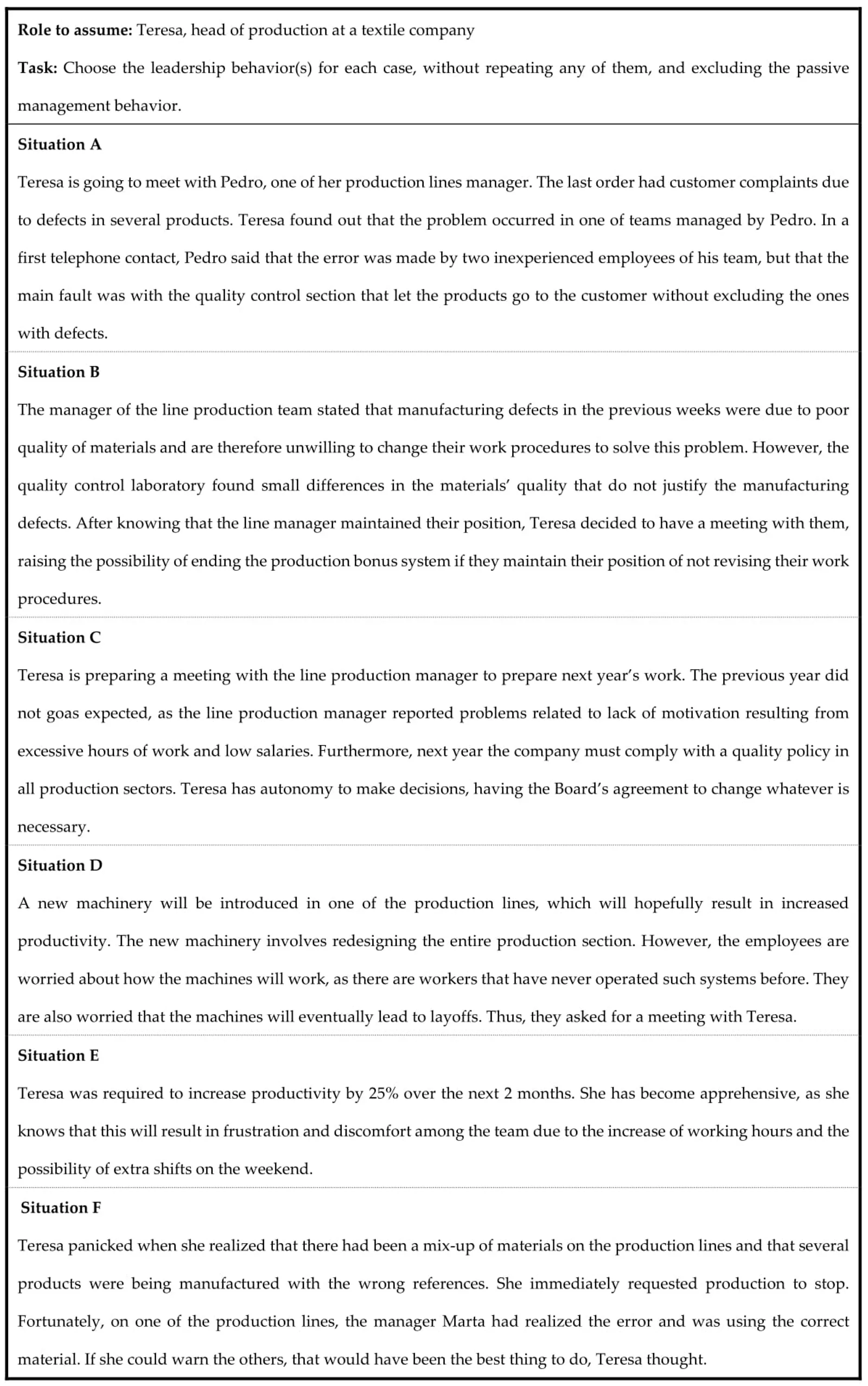
Pro●Leader intervention program: training of leadership behaviors.

After exploring leadership styles and behaviors, participants are asked to reflect on a case study of leadership behaviors in a specific human context. The case study presents six different situations that can hypothetically occur within a work environment. Groups must discuss and select the leadership behaviors most suitable for each situation. Afterward, all groups share and justify their choice with practitioners. Figure 4 illustrates situations for this task, based on work organization. Versions of this case study are also available for sports organizations, educational organizations, health organizations, and security forces organizations. During training, each group selects the version that is most closely related to their professional experience.

The final activity of this part of the program is training. Participants must assume the selected behavior for each situation and contribute to the whole group by using the role-play methodology. The activity follows: (1) three distinct roles are assigned for the three groups (one group of participants assume the leadership behaviors; another group assumes the role of follower of the participant that has adopted the leadership behaviors, i.e., assumes the role of the persons described in the leadership situations; and the third group assumes the role of observer by registering examples of leadership behaviors typified by the participants with the role of leaders); (2) groups have five minutes to prepare for the role-play; (3) role-play begins and practitioners monitor the quality of the interactions and act as a time-keeper (10 min per interaction); and (4) the practitioners ask participants for their opinions about the role-play by canvassing the opinions of leaders, followers, and observers (10 min of reflection).

In sum, after ending this part of the program, participants are trained to identify and adopt leadership behaviors (involved in the Optimal Profile of Leadership) that are more likely to result in a positive outcome. These behaviors will also be later adopted to build the leadership plan for each participant, meaning they will be asked to select the most appropriate behaviors (all or just some of them) to sustain their leadership in a specific situation.

### 3.4. Leadership Plan (Part 3)

**Figure 5:**
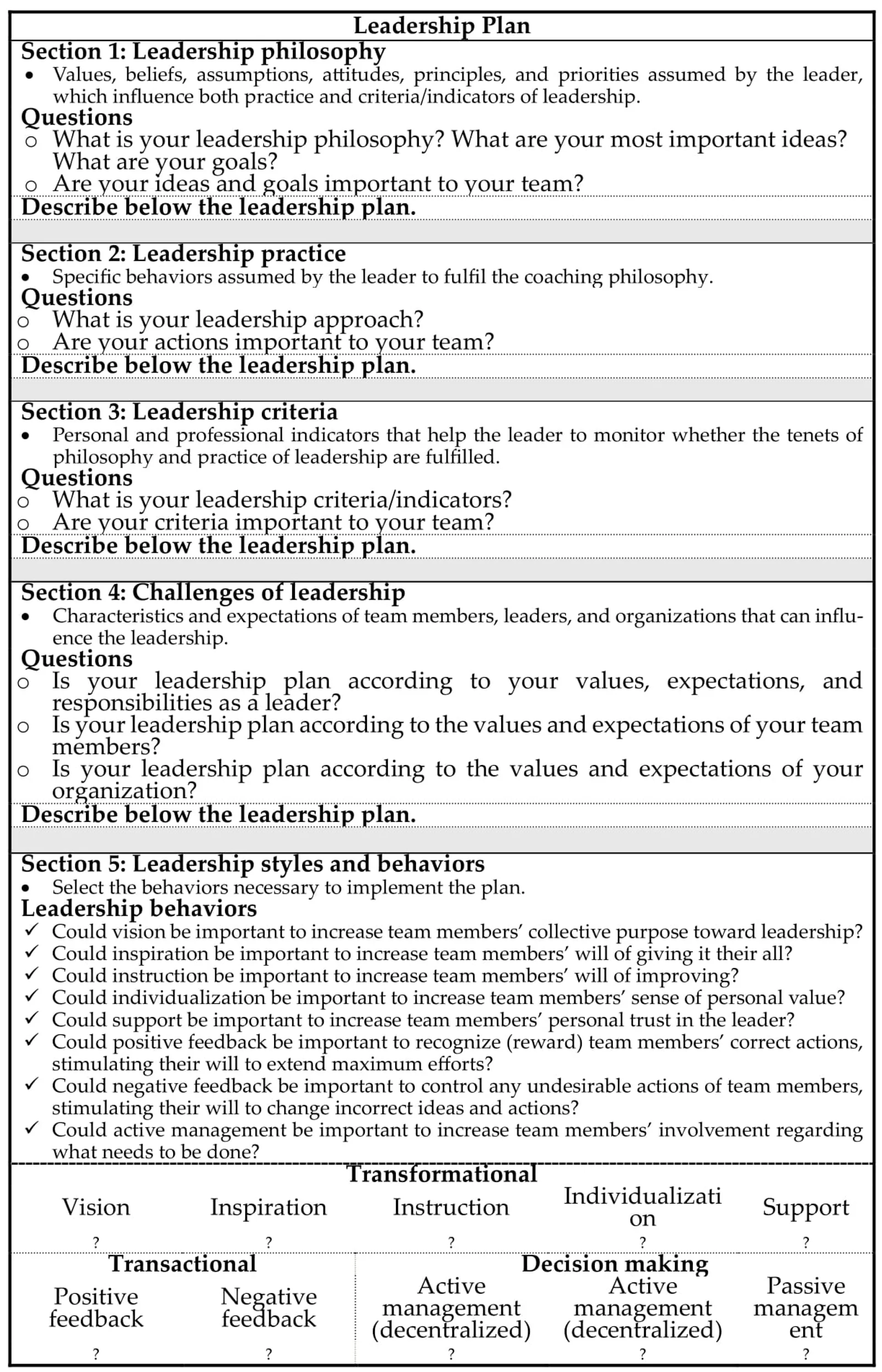
Pro●Leader intervention program: formulating the leadership plan.

Part 3 of the program is dedicated to educating and training participants in formulating leadership cycles. This part begins by explaining the conceptual and practical cycles of leadership as well as the components of the cycles (leadership philosophy, leadership practice, and leadership criteria). Then, participants are asked to formulate their own conceptual cycle of leadership by fulfilling the leadership plan (Figure 5). Participants must describe their leadership philosophy, leadership practice, and leadership criteria, and they must select what specific leadership behaviors are needed to formulate and implement leadership philosophy, leadership practice, and leadership criteria (see Sections 1–3, and 5 of Figure 5). The practitioners observe and give feedback to the participants regarding their plans (which is one of the reasons why there need to be at least two practitioners in the intervention program). The formulation of the conceptual cycle is very challenging to participants because it implies reflecting on their own values, beliefs, principles, and goals as individuals and (potential) leaders, bringing into play aspects related to self-identity, spirituality, and self-concept, among other important psychological constructs (Allen & Fry, 2023; Shamir et al., 2018). This complexity is managed in the program by giving participants the opportunity to first understand what leadership cycles are, and then they are asked to formulate their own leadership cycle as part of the leadership plan, obtaining feedback from practitioners. Later in the program, participants will have the opportunity to practice the leadership plan (which includes the conceptual cycle of leadership), and, once again, they will have more feedback from the practitioners and from other participants (see Section 3.6).

### 3.5. Leadership Plan Adjustment (Part 4)

Part 4 of the program is dedicated to educating and training participants about how antecedent factors of leadership (i.e., leaders’ characteristics, followers’ characteristics, and situational characteristics) can influence the efficacy of leadership (cf. Table 1). Participants are first introduced to the concepts of technical favorability (e.g., the value given by leaders to the mission and tasks to be accomplished and the maturity of team members to accept the mission and execute the tasks), psychological favorability (e.g., the interest and value given by leaders to personal and human aspects of followers and the maturity of followers to accept responsibility inherent to their roles and tasks), and situational favorability (e.g., material, human, and environmental conditions that leaders have to execute their activity) (Fiedler, 1993; Hersey & Blanchard, 1996; Likert, 1967). Then, participants are asked to analyze the need to adjust their leadership plan according to these antecedent factors (see Section 4 of Figure 5). In fact, antecedent factors of leadership can increase the favorability of leadership (i.e., the characteristics of the leader, followers, and situation operate as facilitators to the leader’s activity) or can decrease the favorability of leadership (i.e., the characteristics of the leader, followers, and situation operate as debilitating of the leader’s activity). This implies that participants in the Pro●Leader intervention program analyze how these factors can influence their leadership and then decide if some adjustments should be made in their leadership plan. After deciding if the leadership plan should be adjusted according to the antecedent factors of leadership, participants select the most useful leadership behaviors to increase the favorability of leadership. In this way, the optimal profile of leadership comes again into discussion because participants decide which leadership behaviors are more adjusted to their specific condition. This process strengthens the link between the three major factors of the LEM framework—leadership cycles, leadership styles, and the antecedent factors of leadership—making the program highly tailored to each participant.

### 3.6. Training of the Leadership Plan (Part 5)

Part 5 of the program is dedicated to training the leadership plan by providing a simulated situation to use the practical cycle of leadership by the participants (cf. Table 1). The leadership plan consists of the three elements of the LEM proposal (leadership cycles, leadership styles, and antecedent factors of leadership) being fulfilled by each participant (Figure 5). Specifically, participants choose a certain scenario in their lives where they can exert influence toward others, and then they must elaborate on how to positively influence others, taking into consideration the topics of the Pro●Leader intervention program.

The training of the leadership plan follows the role-play methodology and adheres to the same phases described in the training of leadership styles and behaviors. The only difference is that participants are not training leadership behaviors (as in Part 2), but they are training their own leadership plans. Specifically, it is constituted by the same three roles for the role-play: participants with the role of assuming their own leadership plan, participants with the role of follower of the participant that assumes the leadership plan, and participants with the role of observing the role-play to provide feedback about the way participants assumed their leadership plan. It is important to reinforce that participants may have distinct leadership plans because their context of activity may vary among them. This implies that before starting the role-play, each participant with the role of leader must first give a description of the context, the followers, and the main purpose of the leadership plan, but the rest of the leadership plan cannot be revealed to ensure the subsequent discussions with the followers are realistic and as close as possible to what might happen when participants apply the plan in the future. This procedure is also important for participants in the follower role, as it helps them understand the specific challenges faced by the colleague in the leadership role. This, in turn, helps them interact in a way that reflects what might realistically occur when the leader applies the plan in the near future.

The training provides the opportunity for participants to assume their leadership plans directed for one specific area of their lives (stage of automatization) but also provides the opportunity to discuss with other participants the potential implications of the plan for distinct areas of living (stage of transference). Typically, the formulation and training of the leadership plan represents a valuable task for participants because they can have the opportunity to reflect on how the LEM factors (leadership cycles, leadership styles, and antecedent factors of leadership) can be applied to their own specific situation (i.e., motivation and learning stages). Then, the training of the leadership plan allows participants to assume leadership skills in role-play scenarios while being provided feedback from other participants and practitioners (automatization stage). The exchange of experiences and training of distinct leadership plans between all participants helps each one to comprehend multiple scenarios where leadership can be used, allowing also the possibility of final adjustments to the plans according to the sharing of living experiences between all participants involved in the intervention program (i.e., transference stage).

### 3.7. Other Aspects

Although not critical to the implementation of the program, brief written tests are also used to analyze the acquired knowledge of participants about each part of the program (leadership cycles, leadership styles, and antecedent factors of leadership); more case analyses for a better understanding of the three components of the LEM proposal are also used, and an evaluation system is also included that provides quantitative feedback to participants. In this last case, a Likert scale of four points is used (1 = It can be improved; 2 = Good; 3 = Very good; 4 = Excellent).

## 4. Testing the Pro●Leader Intervention

**Figure 6:**
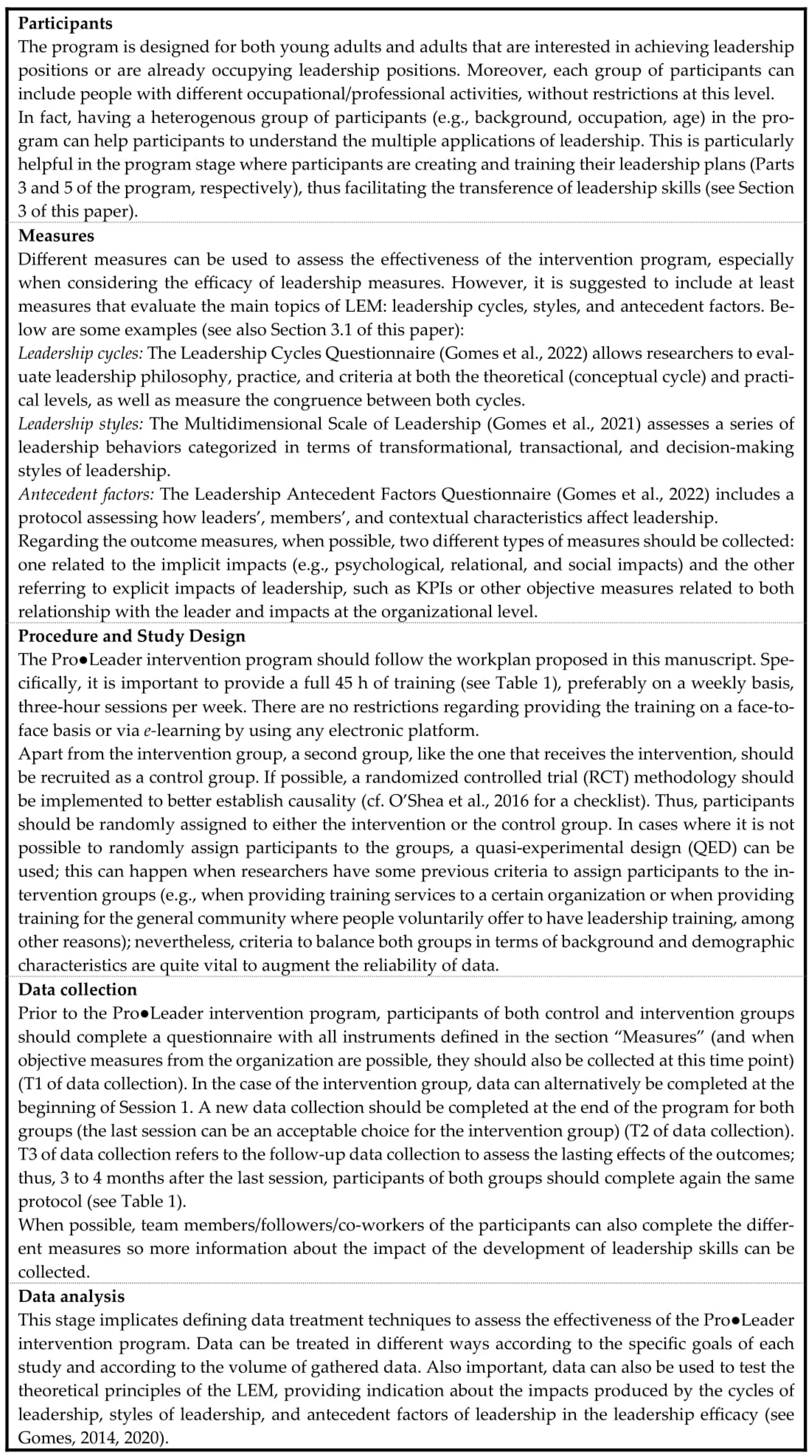
Testing the efficacy of the Pro●Leader intervention program (Gomes, 2014, 2020; Gomes et al., 2021, 2022; O’Shea et al., 2016).

This manuscript describes the Pro●Leader intervention program in detail, which is based on previous literature and, therefore, also provides evidence-based guidelines for developing effective leaders. However, this intervention program still needs to be tested in order to establish its impact on improving leadership skills. Thus, it is important to collect data to verify if the LEM represents a useful approach to explain leadership efficacy and if the three factors included in the model (leadership cycles, leadership styles, and antecedent factors of leadership) represent useful dimensions to stimulate leadership. While there is some encouraging data about the LEM (e.g., Gomes et al., 2022), there are currently no indications related to the efficacy of the intervention. This is quite critical to demonstrate if the intervention is effective and scientifically valid. Figure 6 summarizes the aspects to consider when testing the Pro●Leader intervention program.

The program can represent a useful tool for both professionals interested in stimulating the leadership ability of their clients and for individuals that are (or can be) in a position of leadership. In fact, by mastering the way leadership cycles should be set, how to positively use the leadership behaviors, and how to control the antecedent factors of leadership, participants can have a better perspective of how to modulate the three factors involved in the leadership activity.

## 5. Conclusions

The Pro●Leader intervention program is a leadership training program based on the Leadership Efficacy Model that takes a comprehensive approach to leadership, aiming to respond to some of the recurrent criticism of leadership development programs. Specifically, its innovation relies on the fact that the whole program is centered on the formulation of a leadership plan, which is individualized for each participant, considering their own needs (contradicting the “one size fits all” approach). The formulation of an individual leadership plan implies that participants consider deeply their beliefs, feelings, and assumptions about leadership behaviors when formulating their leadership cycles, which increases the likelihood of change. Moreover, in this process, participants learn different behaviors that can be used to enhance leadership efficacy and adapt to their own circumstances and skills. A third important feature of this intervention program is that it challenges participants to deeply consider the environment in which they expect to exert leadership, namely the characteristics of their followers and organizations, to maximize their efficacy as leaders. Finally, because the program is designed to include the four levels of life skill acquisition (motivation, learning, automatization, and transference), and it does not focus on specific leadership skills but on the process of thinking and developing leadership itself, we believe participants will be more equipped to transfer the knowledge to different contexts, adapting to new situations and organizations (by adapting their individual plans). Of course, as with other programs, the Pro●Leader intervention program assumes a choice about the number of factors that explain the leadership efficacy (leadership cycles, leadership styles, and antecedent factors of leadership), meaning that participants are trained to learn and assume the leadership skills based upon these factors, which does not mean that other factors can adaptively influence leadership. Also of importance, this paper proposes a step-by-step intervention that can help to develop the leadership skills of an interested audience whilst recognizing it is now necessary to collect data to test the effectiveness of the Pro●Leader program.

In a nutshell, the Pro●Leader intervention program aims to respond to the challenge of enhancing leadership with a conceptual and empirical background by implementing three important factors involved in leadership development and efficacy (leadership cycles, leadership styles, and antecedent factors of leadership).

## Figures and Tables

**Table 1:** Pro●Leader Intervention Program: Organization and Areas of Training.

Leadership Efficacy Model	Pro●Leader Program
	**Part 1. Pre-intervention evaluation** **Goal:** Determining the leadership ability of participants before intervention **Duration:** 15 min (before starting session 1)
**Program presentation** **Leadership styles** Transformational leadership Transactional leadership Decision-making leadership	**Presentation** ○ Presenting the program’s goals and main activities ○ Forming the teams of participants **Part 2. Leadership behaviors** **Goal:** Learning and demonstrating positive leadership behaviors (e.g., transformational, transactional, and decision-making) **Duration:** 6 sessions; 3 h per session (18 h)
**Leadership cycles** Leadership philosophy Leadership practice Leadership criteria	**Part 3. Leadership plan** **Goals:** Defining the leadership plan, according to the philosophy, practice, and criteria of participants Selecting the leadership behaviors for the leadership plan (e.g., transformational, transactional, and decision-making) **Duration:** 6 sessions; 3 h per session (18 h)
**Antecedent factors of leadership** Leader characteristics Team members characteristics Situational characteristics	**Part 4. Leadership plan adjustment** **Goal:** Adjusting the leadership plan to the characteristics of the participants, followers, and situation **Duration:** 1 session; 1 h per session (3 h)
	**Part 5. Training of the leadership plan** **Goal:** Assuming the leadership approach in simulated situations **Duration:** 2 sessions; 3 h per session (6 h)
	**Part 6. Post-intervention evaluation** **Goal:** Determining the leadership ability of participants after the intervention **Duration:** 15 min (after last session)
	**Part 7. Follow-up intervention evaluation** **Goal:** Determining the leadership ability of participants after the intervention **Duration:** 15 min (3 to 4 months after last session)
**Total**	Sessions: 15 Duration: 45 h

## Data Availability

No new data were created or analyzed in this study.
